# The Role of Oxidative Stress in Hypertensive Disorders of Pregnancy (Preeclampsia, Gestational Hypertension) and Metabolic Disorder of Pregnancy (Gestational Diabetes Mellitus)

**DOI:** 10.1155/2021/5581570

**Published:** 2021-05-31

**Authors:** Wendy N. Phoswa, Olive P. Khaliq

**Affiliations:** ^1^Department of Life and Consumer Sciences, University of South Africa (UNISA), Science Campus, Private Bag X6, Florida, Roodepoort 1710, South Africa; ^2^Department of Obstetrics and Gynaecology and Women's Health and HIV Research Group, Nelson R Mandela School of Medicine, University of KwaZulu-Natal, Durban, South Africa

## Abstract

*Purpose of the Review*.To highlight the role of oxidative stress in hypertensive disorders of pregnancy (HDP) and metabolic disorders of pregnancy (gestational diabetes mellitus). *Recent Findings*. In both preeclampsia (PE) and gestational hypertension (GH), oxidative stress leads to inadequate placental perfusion thus resulting in a hypoxic placenta, which generally leads to the activation of maternal systemic inflammatory response. In PE, this causes inflammation in the kidneys and leads to proteinuria. A proteinuria marker known as urinary 8-oxoGuo excretion is expressed in preeclampsia. In GDM, oxidative stress plays a role in the pathogenesis of the disease, as a result of over secretion of insulin during pregnancy. This uncontrolled secretion of insulin results in the production of lipid peroxidation factors that also mask the secretion of antioxidants. Therefore, ROS becomes abundant at cellular level and prevents the cells from transporting glucose to body tissues. *Summary*. There is a need for more research investigating the role of oxidative stress, especially in obstetrics-related conditions. More studies are required in order to understand the difference between the pathogenesis and pathophysiology of PE versus GH since investigations on the differences in genetic aspects of each condition are lacking. Furthermore, research to improve diagnostic procedures for GDM in pregnancy is needed.

## 1. Introduction

Oxidative stress has been associated with many diseases including reproductive and pregnancy disorders [1]. Pregnancy is known to increase oxidative stress due to changes in the immune response, which results in high amounts of reactive oxygen species (ROS). The placenta is believed to be the principal source of ROS production during pregnancy [2]. The levels of ROS are normally stabilized by the levels of antioxidants [3]; however, when the levels of ROS exceed that of antioxidants; this leads to oxidative stress [4]. Too much oxidative stress in pregnancy is associated with various conditions that can cause detrimental effects to both the mother and the fetus. These conditions include hypertensive disorders of pregnancy (HDP) and metabolic disorder of pregnancy also known as gestational diabetes mellitus.

Hypertensive disorders of pregnancy (HDP) and metabolic disorder of pregnancy such as gestational diabetes mellitus (GDM) are a major cause of maternal and fetal morbidity and mortality [5–10]. Extensive research has been done to investigate the epidemiology of HDP but it is still enigmatic to date. This epidemic accounts for about 10-15% of maternal deaths worldwide [5, 11]. Hypertensive disorders of pregnancy are composed of the following classes: chronic hypertension, white hypertension, mask hypertension, gestational hypertension (GH), preeclampsia (PE), and haemolysis, elevated liver enzymes, and low platelet count (HELLP) [12].

Several risk factors are associated with HDP, viz., body mass index, anaemia, maternal age, primiparous, multiple pregnancies, previous HDP, gestational diabetes mellitus, preexisting hypertension, preexisting type 2 diabetes mellitus, preexisting urinary tract infection and a family history of hypertension, type 2 diabetes mellitus, and poor diet [13]. The aetiology of HDP remains unknown; however, oxidative stress is speculated to be one of the leading pathophysiological processes of this condition. Several studies have implicated oxidative stress in hypertension [14, 15]. This occurs when there is imbalance of ROS in the vascular walls which causes constriction of blood vessels thus leading to the development of hypertension [16].

Oxidative stress is not only associated with HDP, but also metabolic disorders of pregnancy known as GDM. This occurs when ROS prevents insulin from facilitating cellular glucose uptake. This leads to insulin resistance which subsequently causes GDM.

Therefore, the current review will focus on the role of oxidative stress in HDP (PE and GH) as well as GDM, as these are common conditions in the African population, with preeclampsia being the leading cause of mortality and morbidity in South Africa [17].

## 2. Preeclampsia

Preeclampsia (PE) is a disease of the placenta, which affects both the mother and fetus. It complicates 6-8% of all pregnancies worldwide [18]. Preeclampsia is characterized by new-onset hypertension (systolic blood pressure ≥ 140 and diastolic blood pressure ≥ 90 mm Hg), measured on two occasions every four hours and 24-hour protein urine test of 300 mg/day at ≥20 weeks of gestation or ≥ 1+ proteinuria, detected using a visual dipstick. In the absence of proteinuria, new-onset hypertension can be identified by any of the following conditions: thrombocytopenia (platelet count < 100000 *μ*L), renal insufficiency, HELLP syndrome, pulmonary oedema, or cerebral or visual problems [18]. This disorder remains a serious public health concern in both developed and developing countries contributing to perinatal morbidity and mortality globally [19]. Previous studies have documented that preeclampsia is responsible for 50000-60000 infant deaths per year worldwide [18]. The World Health Organization reported that this multisystem disorder accounts for 16% of maternal deaths in developed countries [20, 21] and 1.8%-16.7% in developing countries such as South Africa, Egypt, Tanzania, and Ethiopia [22]. Initiating events in preeclampsia have been hypothesized to be reduced uteroplacental perfusion as a result of abnormal cytotrophoblast invasion of spiral arterioles [23] which leads to maternal systemic syndrome associated with maternal symptomatic diseases such as hypertension, acute haemolysis, elevated liver enzymes, and low platelets (HELLP syndrome) [24].

### 2.1. Risk Factors of Preeclampsia

The risk factors of preeclampsia are categorized into broad groups, namely, maternal-related factors, paternal-related factors, and gestational factors.

#### 2.1.1. Maternal-Related Factors (Genetic or Acquired)

Familial/genetic factors involve the family history of preeclampsia which increases the risk of the disorder as well as women whose mothers had preeclampsia [25, 26]. Men who have fathered a pregnancy with preeclampsia are more likely to father another pregnancy complicated with preeclampsia with other women [27].

#### 2.1.2. Paternal-Related Factors

Primapatenity hypothesis suggests that risk of preeclampsia is increased among women who have limited exposure to their partner's sperm [28]. Women conceiving following intracytoplasmic sperm injection (e.g., sperm obtained via surgery) have a 3-fold higher risk of preeclampsia compared to women previously exposed to their partner's sperm (e.g., women conceiving after in vitro fertilization or intracytoplasmic sperm injection with sperm obtained from ejaculation) [29].

#### 2.1.3. Gestational Factors

Preexisting medical condition such as long-term hypertension, diabetes mellitus, insulin resistance, obesity, and thrombophilia also attenuate the occurrence and possibility of preeclamptic pregnancies [30, 31].

Furthermore, demographic factors such as maternal age ≥ 35 years and environmental factors such as living at high altitude and stress contribute to the risk of PE [32]. Additionally, smoking and miscellaneous factors such as a large placenta, prolonged pregnancy, placental hydrops, and chromosomal abnormalities also increase the risk of preeclampsia development [33, 34].

### 2.2. Pathophysiology of Preeclampsia

The placenta is primarily associated with the pathophysiology of preeclampsia [35]. Studies have defined preeclampsia as a two-stage model disease in which stage one involves the disturbance of placentation as a result of defective invasion of spiral arteries by cytotrophoblast cells [32, 36]. Stage two involves ischaemia reperfusion of the placenta that causes an increase in cytokines, oxygenation, and reactive oxygen species resulting in increased oxidative stress [23, 37]. This activates the systemic inflammatory response that results in organ-specific changes associated with preeclampsia. Preeclampsia affects various organ systems such as the renal, hepatic, hematological, central nervous system, and cardiovascular system by activating different pathophysiological mechanisms, resulting in hypertension, proteinuria, oedema, clotting, eclampsia, and HELLP syndrome. Even though the aetiology of preeclampsia pathophysiology is still unclear, abnormal placentation has been mentioned to play a principal role in the pathology of preeclampsia [38, 39].

#### 2.2.1. Abnormal Implantation and Vascular Remodelling

The placenta has been defined as the root cause of preeclampsia [37]. The role of the placenta in the pathophysiology of preeclampsia develops as a result of reduced placental perfusion. This is due to failure of the maternal arteries to supply the placenta with nutrients for proper physiological adaptations of a healthy pregnancy [37]. During early stages of implantation, <10 weeks gestation, the embryo is currently at a low-oxygen environment and is not supplied by any maternal vessels but dependent on nutrients supplied by endometrial glands. Placental cytotrophoblast responds to the initial low-oxygen environment with proliferation. At about 10 weeks of gestation, maternal vessels begin to perfuse the embryonic placenta [40]. The placental cytotrophoblast then responds to the subsequent increased oxygen with reduced proliferation and differentiation to an invasive phenotype [41]. Within the uterine wall, cytotrophoblasts deeply invade the spiral arteries then migrate up these vessels and replace the maternal endothelial lining in a retrograde fashion [42].

In a normal pregnancy, the spiral arteries, which are initially small-diameter muscular constricted arteries, dilate strikingly at the decidual end of the vessel [43]. As a result, the spiral arteries attain the physiologic properties that are required to perfuse the placenta adequately [42]. However, in preeclampsia, cytotrophoblast invasion of the interstitial decidual compartment is frequently superficial, and in many locations, spiral artery invasion is incomplete. There are fewer endovascular cytotrophoblasts, and some vessels retain portions of their endothelial and muscular lining, although others are not modified [42, 44]. This then leads to reduced placental oxygenation (placental hypoxia) followed by oxidative stress.

#### 2.2.2. Endothelial Dysfunction

**Figure 1:**
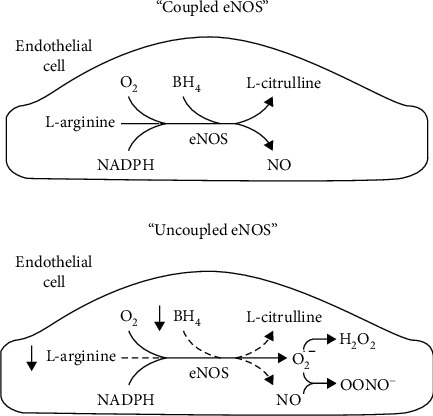
Pathophysiological mechanism of hypertension in preeclampsia, adapted from [37]. Inadequate uterine blood flow to the placenta leads to placental ischemia, which triggers the release of factors such as endothelin-1 (ET-1) and thromboxane (TBX). These factors interrupt endothelial activation factors, namely, nitric oxide (NO) and the prostaglandins (PG_2_), thus increasing angiotensin II sensitivity. This causes a reduction in renal pressure natriuresis and an increase in total peripheral resistance, thus leading to hypertension.

Endothelial dysfunction is characterized by increased endothelin-1, a vasoconstrictor secreted from the endothelial cells. Findings have reported higher plasma concentrations of endothelin of ≈2-3 fold in women with preeclampsia [38, 45]. Mechanisms responsible for systemic maternal vascular dysfunction remain undefined. A decrease in vasodilators such as nitric oxide and prostacyclin and an upregulation of endothelin, thromboxane, superoxide, and increased vascular sensitivity to angiotensin II have been constantly shown to play a role in the development of hypertension by impairing renal function as well as increasing total peripheral resistance [23, 37, 46, 47].  Figure 1 below represents the pathophysiological mechanism of preeclampsia.

### 2.3. Oxidative Stress in Preeclampsia

The pathophysiology of PE remains undefined and is characterized by a two-stage model: stage 1 which is characterized with poor trophoblast invasion and poor spiral artery remodelling and stage 2 (the maternal syndrome) during which the patient develops a systemic inflammatory reaction with generalized endothelial dysfunction. Oxidative stress occurs at each stage of this model and could be the “common denominator” for all forms of PE [48]. Under normal placentation, conditions (stage 1), ROS behaves like second messengers and is responsible for various cellular responses such as proliferation, migration, and angiogenesis. During the process of cellular responses, ROS is rapidly captured (by antioxidants such as vitamins C and E) or metabolized by multiple systems of antioxidant defenses (for example, superoxide dismutases (SOD), glutathione peroxidases (GPx), catalase, thioredoxins, and peroxyredoxins). However, when the production of ROS exceeds the capabilities of the antioxidant defense systems, oxidative stress begins, thus resulting to inflammatory responses or preeclampsia development [48]. An increase in the markers of oxidative stress has been observed in women with preeclampsia and small-for-gestational-age infants [3, 49].

The association between preeclampsia and oxidative stress has also been demonstrated in animal studies [50, 51]. A study by Beauséjour et al. determined whether maternal disturbances in pregnancy lead to placental oxidative stress. This was done by increasing sodium intake in drinking water of Sprague-Dawley pregnant rats. Oxidative stress markers were found to be increased in sodium-supplemented rats compared to nonsupplemented rats [52]. Another rat model study indicated that high blood pressure and sympathetic overactivity in preeclamptic rats is associated with increased ROS via upregulation of NADPH oxidase subtype (NOX4) expression in the rostral ventrolateral medulla [53].

Since oxidative stress is characterized by increased production of ROS and toxic lipid peroxides, particularly for endothelial cells [54], various mechanisms are involved in the production of ROS.

#### 2.3.1. Mechanisms Involved in Reactive Oxygen Species (ROS) Production

The production of ROS is modulated by antithrombin-1 and TNF-*α* whose generation increases during PE [55]. Presence of ROS in maternal circulation activates monocytes and neutrophils, which produce proinflammatory cytokines, such as TNF-*α* and IL-6, antiangiogenic factors, and microparticles [37]. Activated neutrophils produce ROS by the action of several enzymes, including NADPH oxidase [56], xanthine oxidase (XO), and the decoupling of eNOS (endothelial nitric oxide synthase) [57]. In preeclamptic patients, peripheral circulation of neutrophils has been reported to be significantly high. Therefore, neutrophils have been identified as the principal site for ROS production during preeclampsia [58].

NADPH oxidase is a ubiquitous enzyme, highly expressed in neutrophils and endothelial cells [46]. The moderate doses of ROS produced by NADPH oxidase participate in the regulation of vascular tone [56]. However, NADPH oxidase activity is increased during PE [59, 60], suggesting a role for this enzyme and for neutrophils in the pathogenesis of oxidative stress during this pathology. NADPH oxidase generates the superoxide anion which, among its different actions, acts by decoupling NOS (or nitric oxide synthase), which generates NO whose combination with the superoxide ion forms peroxynitrite (ONOO -) [56].

**Figure 2:**
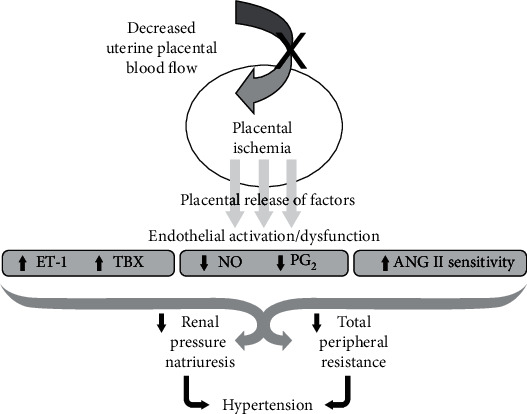
Schematic representation of “uncoupling” of nitric oxide (NO) synthesis, adapted from [70]. Suboptimal concentrations (↓) tetrahydrobiopterin (BH4) are required for “uncoupling.” Superoxide anion: O; hydrogen peroxide: H_2_O_2_; peroxynitrite anion: OONO^−^; endothelial NO synthase: eNOS.

Nitric oxide (NO) is the body's most ubiquitous second messenger which plays a pivotal role in vascular functions such as endothelium-dependent dilation [61], angiogenesis [62], and leukocyte adhesion [63]. Nitric oxide has been reported to have a significant importance during normal placental development by facilitating trophoblast invasion [64]. Nitric oxide also participates in placental vasculogenesis and angiogenesis through VEGF and angiopoietin signaling molecule in order to maintain adequate fetomaternal blood flow [65]. However, under abnormal conditions, NO bioavailability is altered. This alteration is thought to be due to the uncoupling of endothelial nitric oxide synthase (eNOS). Endothelial nitric oxide synthase is an enzyme responsible for the regulation of vascular endothelial cell function through production of NO. Bioavailability of eNOS is also affected by multiple factors, including increased oxidative stress, which results in uncoupling of eNOS with subsequently less NO and more superoxide generation (Figure 2). This occurs due to the enhanced oxidation of BH4 (tetrahydrobiopterin) which in turn leads to a decrease in BH4 bioavailability. As a pivotal factor, BH4 is necessary for optimal eNOS activity which facilitates NADPH-derived electron transfer from eNOS reductase to the oxygenase domain to convert L-arginine to NO and L-citrulline. Therefore, when BH4 levels are inadequate, eNOS becomes unstable and uncoupled, leading to subsequently less NO production and more superoxide generation followed by oxidative stress and endothelial cell death or endothelial dysfunction [66, 67]. Endothelial dysfunction and reduced NO bioavailability have been shown to play a major role in the pathophysiology of preeclampsia. Inadequate NO has been reported to play a role in the pathogenesis of preeclampsia [68, 69]. Figure 2 below is a schematic representation of the uncoupling of nitric oxide synthesis.

## 3. Oxidative Stress in Gestational Hypertension

Another hypertensive disorder of pregnancy that is similar to preeclampsia is gestational hypertension (GH). Gestational hypertension is defined as pregnancy-induced hypertension without proteinuria [71, 72]. Both PE and GH oxidative stress are noted as the primary factors that lead to the pathogenesis and pathophysiology of each disease [72]. A study by Kurlak et al., which investigated oxidative stress profiles in hypertensive states (PE and GH), indicated that oxidative stress profiles do not differ in hypertensive states [71]. The only difference between PE and GH is that in PE there is proteinuria [72]. We speculate that this proteinuria occurs as a result of oxidative stress in the kidneys. A previous study documented that urinary oxidative stress marker, known as urinary 8-oxoGuo excretion, is associated with albuminuria [73] which is a risk factor marker for early kidney dysfunction. Albumin is a protein that is found in the blood and in preeclamptic patients; this protein is excreted in urine, and this leads to a condition known as proteinuria which is a characteristic of preeclampsia [74]. Interestingly, urinary 8-oxoGuo excretion has also been linked to cardiovascular mortality risk in patients with diabetes mellitus [75, 76].

GH, just like in PE oxidative stress, emanates when the production of ROS exceeds the antioxidant capacity resulting in overall damage to cells [2]. Although the mechanism behind manifestation of both GH and PE is similar, more studies investigating differences in genetics aspects of each condition are needed.

## 4. Gestational Diabetes Mellitus (GDM)

Gestational diabetes mellitus (GDM) is described as carbohydrate intolerance with onset or first recognition during pregnancy [77], resulting in hyperglycaemia and hyperinsulinaema [78]. It commonly occurs in the second trimester of pregnancy [79]. GDM usually disappears after the delivery of the baby, but predisposes women and babies to the development of type 2 diabetes [80]. Higher risk is associated with a possible unrecognisable presence of type 1 or type 2 diabetes [81, 82]. Type 2 diabetes (T2D) is a condition manifested by different genetic factors, lifestyle, and obesity resulting in insulin resistance. T2D prevalence has risen from 4.7% in 1980 to 8.5% in 2014 [83, 84]. Type 1 diabetes is described as the inability of insulin production and secretion by pancreatic *β*-cells, resulting in autoimmune destruction. This makes the body insulin dependent, and individuals diagnosed with type 1 diabetes take insulin injections daily. Unfortunately, at this point in time, insulin can only be administered by injection since it is synthesized from proteins. Ingestion of proteins may lead to their denaturation; therefore, insulin is inactivated [81]. Type 1 diabetes is normally diagnosed in children and young adults, and it affects only about 5% of the world's diabetes population [85].

### 4.1. Prevalence

The prevalence of GDM is dependent on maternal age, BMI, and ethnicity. Globally, GDM affects 1-28% in all pregnancies [86]. In the United States, GDM affects 5% of pregnancies; 3-7% are affected in the United Kingdom and 2-6% in other European countries. Higher disease prevalence was observed in African, Asian, Indian, and Hispanic women [87, 88]. According to 2011 review reports, 0-9% of the pregnant population is affected in sub-Saharan Africa [89]. In South Africa, GDM accounts for 1.6-8.8% of pregnancies [90].

### 4.2. Risk Factors

Women at higher risk of developing GDM include those with a family history of diabetes in first-degree relatives, obesity, body mass index (BMI) ≥ 30, maternal age > 25 years old, have had spontaneous abortions, previous adverse pregnancy outcome (congenital abnormality, miscarriages, and stillborn births), macrosomic deliveries, constant glycosuria, and proteinuria, as well as GDM in their previous pregnancies [90].

### 4.3. Pathophysiology of Gestational Diabetes Mellitus

The pathophysiology of GDM is associated with insulin resistance (IR) and metainflammation. During normal pregnancy, IR levels rise slightly (40-50% above normal) but are recompensed by insulin secretion of 200-250% for normal glucose management [91, 92]. Gestational diabetes development is still unclear to date but suspected to be due to *β*-cell dysfunction [93]. If the pancreatic-*β*-cells are unable to regulate the elevated amount of insulin released during pregnancy, GDM may occur [93]. Researchers have reported that GDM is moderate in patients with a regular *β*-cell response compared to those with *β*-cell dysfunction [94, 95]. Beta-cell dysfunction may be caused by an autoimmune *β*-cell destruction due to anti-islet antibodies and/beta-cell antigens in circulation. This condition resembles that occurring in type 1 diabetes [94, 96, 97]. A defective *β*-cell dysfunction may also arise from mutations that transpire in autosomes, an autosomal dominant inheritance pattern known as maturity-onset diabetes of the young (MODY). Interestingly, MODY has been found to develop in different categories in women with GDM, with MODY-2, involved in mutations of the glucokinase gene, MODY-3, characterised by a genetic mutation in hepatocyte nuclear factor 1*α*, and lastly, MODY-4, involved in a mutation in insulin promoter factor 1 [94, 96–99]. All these mutations are suspected to be asymptomatic and occur in preexisting diabetes, with recognition during glucose screening in pregnancy [77].

Another factor suspected to play a role in GDM development is inflammatory response. In normal pregnancies, it is transformed and may result in maternal complications such as premature deliveries, gestational hypertension, preeclampsia, and gestational diabetes mellitus if response is highly expressed. Increased inflammatory response can be caused by obesity. Women with a high body mass index (BMI) have increased cytokine release in circulation compared to those with a normal BMI [100]. Interleukin-6 (IL-6), IL-8, IL-1*β*, tumor necrosis factor-*α* (TNF-*α*), and C-reactive protein (CRP) were recently found to be elevated in pregnant obese women than in pregnant women with a normal body weight [93]. Obesity is then suggested to alter proinflammatory and anti-inflammatory cytokine expression since inflammatory cytokines in circulation are synthesized by adipose tissue, resulting in IR [92]. Inflammation may also result from oxidative stress [101].

### 4.4. Oxidative Stress in Gestational Diabetes Mellitus

Oxidative stress arises from an imbalance between prooxidants and antioxidants in a cell [102]. Prooxidants are reactive oxygen species (ROS) which express free radicals and nonradical derivatives of oxygen [93]. Examples of ROS can be the following: hydrogen peroxide (H_2_O_2_), superoxide anion (O_2_^−^), hydroxyl radical (OH), organic hydroperoxide (ROOH), and more [102]. Women with GDM have been reported to have a higher free radical production in circulation than those without GDM. ROS has been shown to result in endothelial dysfunction due to increased production of cytotoxic oxidative stress [103, 104].

The placentae of women with GDM show a higher release of 8-isoprostane compared to placentae of normal pregnancies [105]. In addition, a positive correlation was found between 8-isoprostane and plasma glucose, indicating that lipid peroxidation is associated with glycaemic regulation [105]. Interestingly, Lappas et al., who found that the placentae of women with GDM showed a decrease in response to oxidative stress, found confounding results. The placentae of GDM and normotensive women were exposed to oxidative stress, and the results showed a twofold increase of 8-isoprostane release in normotensive placentae while no change was observed in GDM placentae [102]. This was suspected to be due to elevated antioxidants in the placenta that accumulated during mild oxidative stress that occurs during the course of pregnancy.

**Figure 3:**
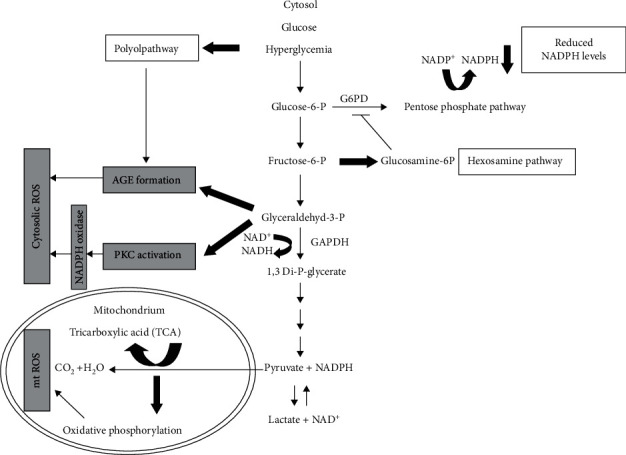
Schematic diagram showing how hyperglycaemia leads to oxidative stress, adapted from [102]. Hyperglycaemia is shown to be the prominent cause of oxidative stress in women with GDM. This occurs due to an increase in superoxide anion production in the mitochondria, caused increased pyruvate, and NADH formation, which activates various pathways. These include the polyol pathway, hexosamine pathway, activation of protein kinase C, and the activation of advanced glycation end products (AGE). All these pathways lead to an elevation in ROS.

Antioxidants can either be obtained from diet or produced within the cell and can be water or lipid soluble, and water soluble antioxidants are substances such as vitamin C, lipoic acid, uric acid, and glutathione and can react with water in the cytosol [102]. Lipid antioxidants provide a protective mechanism against lipid peroxidation in the cell, including vitamin E, carotenes, and ubiquinol (coenzyme Q). Increased levels of lipid peroxidation are found in individuals with reduced glycaemic regulation [106–108]. The process is illustrated in Figure 3 below.

## 5. Conclusion

Oxidative stress plays a central role in the pathogenesis and pathophysiology of hypertensive disorders of pregnancy. Clear evidence indicates its involvement in the initiation and progression of these disorders. Therefore, factors that lead to the production of ROS (antithrombin-1 and TNF-*α*); should be further investigated and may be used as potential biomarkers for the early detection of oxidative stress in pregnancy.

## 6. Future Studies

Future aspects regarding GDM have been implemented based on genotyping since it is believed that mutations in genes may be associated with defects in beta-cell functioning and subcellular insulin signaling, thus leading to GDM. Additionally, the genes responsible for the activation of antithrombin-1 and TNF-*α* should be sequenced to identify mutations that may be associated with the possible overexpression of these factors, leading to the uncontrolled production of ROS in women with hypertensive disorders of pregnancy.
